# A review of implementation and evaluation frameworks for public health interventions to inform co-creation: a Health CASCADE study

**DOI:** 10.1186/s12961-024-01126-6

**Published:** 2024-03-28

**Authors:** Giuliana Raffaella Longworth, Kunshan Goh, Danielle Marie Agnello, Katrina Messiha, Melanie Beeckman, Jorge Raul Zapata-Restrepo, Greet Cardon, Sebastien Chastin, Maria Giné-Garriga

**Affiliations:** 1 Faculty of Psychology, Education and Sport Sciences, Universitat Ramon Llul, Blanquerna, Barcelona, Spain; 2 Department of Physical Activity and Sport Sciences, FPCEE Blanquerna, Universitat Ramon Llull, Carrer del Císter, 34, 08022 Barcelona, Spain; 3 Department of Public and Occupational Health, Amsterdam Public Health Research Institute, Amsterdam UMC, Vrije Universiteit Amsterdam, Meibergdreef 9, 1105 AZ Amsterdam, The Netherlands; 4 School of Health and Life Sciences, Glasgow Caledonian University, Cowcaddens Road Glasgow, Scotland, G4 0BA UK; 5 Department of Movement and Sports Sciences, Physical Activity and Health, Ghent University and Policy Research Center Sport, Krijgslaan 281 - S2, 9000 Ghent, Belgium; 6 School of Psychology, Artevelde Hogeschool, Ghent, Belgium

**Keywords:** Implementation, Evaluation, Framework, Public health, Co-creation, Evidence-based, Review, Systems thinking

## Abstract

**Background:**

By including the needs and perspectives of relevant stakeholders, co-creation is seen as a promising approach for tackling complex public health problems. However, recommendations and guidance on how to plan and implement co-creation are lacking. By identifying and analysing existing implementation and evaluation frameworks for public health, this study aims to offer key recommendations for professional stakeholders and researchers wanting to adopt a co-creation approach to public health interventions.

**Methods:**

Firstly, PubMed and CINAHL databases were screened for articles introducing original implementation and evaluation frameworks for public health interventions. Backwards snowballing techniques were applied to the included papers. Secondly, identified frameworks were classified and relevant data extracted, including steps and constructs present in the frameworks. Lastly, recommendations were derived by conducting thematic analysis on the included frameworks.

**Results:**

Thirty frameworks were identified and data related to their nature and scope extracted. The frameworks’ prominent steps and constructs were also retrieved. Recommendations related to implementation and evaluation in the context of co-creation were included.

**Conclusion:**

When engaging in co-creation, we recommend including implementation considerations from an early stage and suggest adopting a systems thinking as a way to explore multiple levels of influence, contextual settings and systems from an early planning stage. We highlight the importance of partnering with stakeholders and suggest applying an evaluation design that is iterative and cyclical, which pays particular attention to the experience of the engaged co-creators.

**Supplementary Information:**

The online version contains supplementary material available at 10.1186/s12961-024-01126-6.

## Background

Implementation science has been defined as the transfer of clinical research findings and evidence-based results into the real world and hence how a study can affect or hinder its uptake in the routine practice [1–4]. Thus, implementation science is set to observe and study the gap between, on one side, a solution developed in a controlled environment and, on the other, the specific context where the intervention is applied by looking at contextual factors that may act as barriers or facilitators.

However, interventions and solutions built in a controlled setting and transferred to specific context, have been argued to obtain limited success, mostly in the long term. For instance, the misconsideration for complex systems and factors related to settings and the targeted population have been said to influence the lack of effectiveness [5, 6]

Taking into account the relevance and inclusion of stakeholders’ knowledge in research production as been put forward as a possible way to address the research-practice gap [2, 3]. For this reason, more recently, implementation science has been experiencing a shifts from this type of linear and controlled production models to more iterative participatory and complex models [7–9] with the design and creation of solutions and interventions directly in the real world.

Involving relevant stakeholders from the earliest stage of intervention design and/or implementation has been considered a way to increase uptake and positively affect not only patient satisfaction but also the quality of the service [10–15]. In line with this considerations, co-creation has been brought to the forefront as an opportunity to increase the successful uptake of evidence-based interventions and practices through meanginful and deep engagement of key stakeholders [16–19].

Co-creation is a collaborative approach of creative problem solving engaging diverse stakeholders at all project stages, from determining and defining the problem through to the final stages of a project [20]. By facilitating collaboration among key stakeholders, co-creation aims to taking into account social determinants and contextual factors that may influence the intervention’s feasibility and acceptability from the earliest stage of intervention design.

Considering co-creation’s intention to work within real-life settings and conditions, systems thinking seems to be a valuable approach to explore and potentially adopt when designing and evaluating co-creation. Adopting a system thinking approach would allow assessing contextual elements from an early stage of the intervention and gathering considerations around systemic factors that may influence the public health issue [21].

The need for formative evaluation in co-creation has been argued to be crucial to co-creation processes. An evaluation is intended to be formative when the implementation team and/or staff use data to improve or adapt the process of implementation [1]. Van Dijk-de Vries [22] argues that researchers, when co-creating, should assess the stakeholders’ engagement to ensure their perceptions are captured, suggesting this happens throughout the implementation. Formative evaluation would enable, if needed, to adapt and adjust the intervention.

Despite research advancement in the field, implementation guidance and recommendations for the planning and implementation of co-creation processes are lacking as existing implementation and evaluation frameworks have not been designed specifically for such approaches. The need to develop dedicated implementation and evaluation guidelines for co-creation lies in the distinctive nature of co-creation approaches, involving collaborative efforts with diverse stakeholders, emphasizing shared decision-making, innovation and creativity.

Implementation and evaluation guidance needs to be further developed to address the dynamic and participatory nature of these processes and the unique challenges of fostering meaningful partnerships, navigating diverse perspectives and harnessing collective creativity. Closing this gap is essential not only for the successful implementation of co-creation initiatives but also for unlocking the full potential of these collaborative efforts within the broader landscape of public health interventions. This study aims to address this gap by reviewing existing frameworks and offering an overview of recommendations that may guide the design and implementation of co-created interventions.

## Methods

### Eligibility criteria

To be included, studies had to describe or introduce an implementation or evaluation framework, therefore describing or introducing a framework representing key stages, factors, constructs or variables that explain or influence the implementation and/or evaluation of programs/interventions. Frameworks had to be generalizable, and therefore designed to be applicable for all public health topics. For the scope of this review, an intervention was defined as a set of actions with a coherent objective to bring about change or produce identifiable outcomes [26]. We identified the Promoting Action on Research Implementation in Health Services (PARIHS), designed in 1998, as our start date search, as one of the first frameworks to make explicit the multi-dimensional, complex nature of implementation and highlight the central importance of context [27]. Table 1 includes further details on applied criteria.

**Table 1:** Eligibility criteria

Inclusion criteria	
Criterion	Definition
Framework	Study describing or introducing a framework representing key stages, factors, concepts or variables that explain or influence the implementation and/or evaluation of programs/interventions.
Scope:	Study/paper complied with one of the following definitions of implementation and evaluation frameworks:
Process model	Specifying steps (stages, phases) in translating research into practice, including the implementation and use of research. Process models aim to describe and/or guide the process of translating research into practice. Included action models is a process model that provides practical guidance in planning and executing implementation endeavours and/or strategies to facilitate implementation.
Determinant framework	Specifying (also known as classes or domains) of determinants and individual determinants, which act as barriers and enablers (independent variables) that influence implementation outcomes (dependent variables). Frameworks that aim to understand and/or explain influences on implementation outcomes.
Evaluation framework	Specifying aspects of implementation that could be evaluated to determine implementation success. This can include programme, outcome and/or process evaluation frameworks.
Generalizability	Relevant to public health in its broadest sense and/or designed for all public health interventions. Studies do not have to be specifically designed for co-creation.
	If applied to a specific healthcare setting, introducing a general framework designed for all types of interventions.
Intervention	Intending interventions as actions that aim for a change in practice within public health [61].
Publication date	Studies that were conducted between 1998 and 2022.
Excluded criteria	
Generalizability	If introducing frameworks designed especially for a specific type and/or setting of an intervention. If presenting a protocol of a framework.
	If focus was on organizational context.
	If referring to a quality improvement framework.
	If referring to the training of students/practitioners during implementation.
Focus	If focussed only on a specific domain, factor or strategy.

### Search strategy

This scoping review followed the Preferred Reporting Items for Systematic Reviews and Meta-Analysis (PRISMA) guidelines for producing the PRISMA flow diagram (see Additional file 1). A specialist librarian was involved in developing the search strategy on PubMed and CINAHL. These were equal search strategies adapted for each database. The detailed search is available in Additional file 2. In addition, we applied a snowballing technique of pursuing backwards references cited in selected publications.

### Process of selection

At least two authors were independently involved in all review studies through each stage [23]. D.A., K.M., J.Z., M.B., M.G. and G.L. were involved in the title and abstract screening. All the latter co-authors, with the addition of K.G., conducted the full-text screening. D.A., K.M., J.Z., M.B., M.G., G.L. and K.G. took part in the data extraction, and K.G. and G.L. conducted the data analysis. Authors conducted individual and independent reviews through the software Rayyan.^1^ Discrepancies were resolved by consensus and, if unresolved, by the involvement of a third reviewer (M.G., G.L., G.C.).

**Fig. 1:**
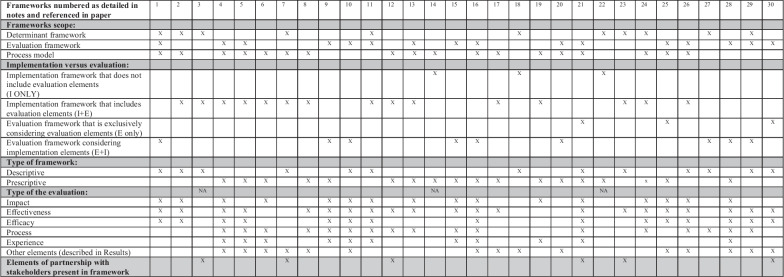
Classification of extracted frameworks

Included frameworks are reported in Fig. 1. From each framework, we extracted prominent constructs (Additional file 3) and steps (Additional file 4). For extraction purposes, we defined constructs as a fundamental unit of thought, smaller than a judgment or theory but integral to them [24]. Identified steps were presented by categorizing them into the Leask’s et al. [25] framework included stages of planning, conducting, evaluating and reporting [25].

### Data extraction

An Excel template was developed to extract information relate to the framework’s nature, type and scope, positioning of implementation and evaluation considerations within the frameworks, and type of evaluation covered and other elements (see Table 2 for full list of data extracted). For each framework, if applicable, we extracted the main constructs (Additional file 3) and main phases (Additional file 4). The data extraction sheet was developed and piloted by two reviewers (G.L., M.G.) on two eligible papers and reviewed accordingly. D.A., K.M., J.Z., M.B., M.G., K.G. and G.L. extracted all data independently and blind-folded.

**Table 2:** Data extracted

Data	Definition
Implementation and/or evaluation framework	Implementation framework that does not include evaluation elements.
	Implementation framework that includes evaluation elements.
	Evaluation framework that is exclusively considering evaluation elements.
	Evaluation framework considering implementation elements.
Scope	Process model, as defined in eligibility criteria.
	Determinant frameworks, as defined in eligibility criteria.
	Evaluation framework, as defined in eligibility criteria.
Framework type	Descriptive, i.e. describing properties, characteristics and/or qualities [59].
	Prescriptive, providing direction on the sustainability process via a series of steps or procedures [59].
Type of evaluation	Impact evaluation, defined as the extent to which an intervention has had the right effect and is working in achieving its set goals [60].
	Effectiveness, the performance of intervention in a real-world context and setting [57].
	Efficacy, understood as the evaluation of an intervention performing under ideal and controlled circumstances [57].
	Process, intended as the consideration for why an intervention has worked, failed or had unexpected consequences. It can be used to assess fidelity and quality of implementation, explore contextual factors and clarify causal mechanisms [34].
	Experience, through which this study intends the evaluation of experiencing the intervention as a stakeholder, participant or user.
	Other subjects of evaluation described in the section below “the role of evaluation in implementation frameworks”.
Steps	Steps and phases included as part of each framework that are essential as part of the framework.
Constructs	Fundamental unit of thought, smaller than a judgment or theory but integral to them [24].

### Data analysis

With the scope of aligning findings and come to a final set, extracted data was then cross-checked between G.L. and K.G. G.L. and K.G. sought consensus and, if in disagreement, they involved MG as a third reviewer. Final data was then plotted by G.L. in Fig. 1, which includes the framework’s classification data and steps and constructs reported in Additional files 2 and 3. K.G. and G.L. further conducted thematic analysis of included frameworks, as described below.

### Thematic analysis

Through thematic analysis, we aimed to identify from included frameworks recommendations that would be relevant to the context of co-creation. This process followed the six stages as outlined by Braun and Clarke [58], including the following steps: (1) G.L. and K.G. familiarized with the data and wrote familiarization notes; (2) G.L. and K.G. developed a coding; (3) G.L. and K.G. independently generated initial themes from coded and collated data; (4) finally met to develop and reviewing themes; as well as to (5) refine, define and name themes; and (6) they applied the thematic framework to the remaining frameworks. [24]. The two researchers (G.L. and K.G.) independently coded four frameworks and then met to develop the thematic framework, which was then applied to the remaining frameworks. The themes and related recommendations reported in the results section emerged as the result of the data coding and iterative theme development [58].

Coding themes included the following: (a) early implementation considerations—exploring how the frameworks were including early implementation consideration; (b) system thinking—understand in what way frameworks were framed within a systems thinking paradigm [21, 29, 30]; (c) partnering with stakeholders—retrieving information on how the frameworks partnered with the stakeholders; (d) experience—how frameworks were pointing towards an assessment of the users’ experience throughout the process; or, finally, (e) iterative and cyclical evaluation—we explored how frameworks were accounting for this aspect.

## Results

### Summary of identified frameworks

From a total of 9061, after removing duplicates, 5284 papers were screened at title and abstract and 425 retrieved for full-text screening. We identified 30 articles and related implementation and evaluation frameworks (Table 3).

**Table 3:** List of frameworks

First author(s)	Short title/name of framework	Aim
Bednarczyk et al. [76]	Practice—provider—patient (P3) model	Provide a framework for the development, implementation and evaluation of preventive care promotion interventions.
Best et al. [43]	Integrative framework for community partnering	Help to understand the interplay among individual-, family-, organizational-, and community-level factors.
Cambon and Alla [33]	Intervention system theory	Support with evaluating the interventional system within a theory-driven paradigm.
Campbell et al. [31]	Framework for designing and evaluating complex interventions	Support with the design of randomized controlled trials of complex interventions.
Carroll et al. [45]	A conceptual framework for implementation fidelity	Support with measuring implementation fidelity and understanding its place in the process of intervention implementation.
Chen [38]	The bottom-up approach to integrative validity: a new prospective for program evaluation	Offers a model for program evaluation and improved validity.
Craig et al. [39]	The medical research council guideline	Offers guidance for developing and evaluating complex interventions.
Damschroder et al. [36]	The consolidated framework for implementation research (CFIR)	Guide systematic research that supports rapid-cycle evaluation of the implementation of health care delivery interventions.
Eslava-Schmalbach et al. [37]	Equity-focussed implementation research for health programs; EquIR	Reduce or prevent the increase of existing inequalities during implementation.
Glasgow et al. [42]	The reach, effectiveness, adoption, implementation and maintenance (RE-AIM) framework	Guide the planning and evaluation of programmes.
Gonot-Schoupinsky and Garip [46]	FRAME-IT	Support the planning and design of early-stage interventions, including constructs such as feasibility, acceptability and tailorability.
Green and Kreuter [34]	The PRECEDE-PROCEED model	Support the assessment of health needs for designing, implementing and evaluating health promotion and other projects.
Gurewich, Garg, and Kressin [32]	The objectives, audience/insight, strategy/ideas (OASIS) framework	Map the known and hypothesized pathways by which unmet social need screening and referral interventions may impact outcomes.
Hennessey Lavery et al. [37]	The Community Action model	Provide communities with a framework to acquire the skills and resources to plan, implement and evaluate health-related actions and policies.
Hyner [77]	A procedural model for planning and evaluating behavioural interventions	A model for planning, implementing and evaluating health behaviour change strategies.
Jolley et al. [50]	Framework for planning and evaluating community participation, collaborative partnership and equity	Support the assessment and evaluation of community participation, collaborative partnership and equity.
Kitson et al. [40]	The PARIHS framework	Guide research implementation by looking into evidence, context, and facilitation.
Leask et al. [25]	PRODUCES framework	Identifies key principles and recommendations for co-creation public health interventions.
Lo and Karnon [41]	In-DEPtH framework	Support health agencies to commission services that are evidence-based, contextually relevant and stakeholder engaged.
Marckmann et al. [35]	Putting public health ethics into practice: a systematic framework	An ethical framework to guide professionals in planning, conducting, and evaluating PH interventions.
Masso et al. [47]	Evolution of a multilevel framework for health program evaluation	Guide the evaluation of health programmes.
Michie et al. [78]	The behavioural change wheel	Guide and improve the design and implementation of evidence-based behaviour change interventions.
MMWR [54]	Framework for programme evaluation in public health	To guide public health professionals in their use of programme evaluation.
Nguyen et al. [79]	Scale-up readiness assessment framework	Guide the process of scaling of a population health intervention.
O’Connor-Fleming et al. [47]	A framework for evaluating health promotion programmes	To support practitioners with the evaluation of health promotion programmes.
Racher and Annis [48]	The community health action (CHA) model	To guide the community assessment, planning, implementation and evaluation of the process.
Titler [51]	Translation Science and Context	A guide to help assess context in translation science.
Wilson et al. [49]	The knowledge to action framework	Provide support for the identification of decision points, interactions, and supporting structures to enable to move knowledge to sustainable action.
Zucca et al. [52]	Assessment framework for pre-implementation policy evaluation	Supporting the recognition of key mechanisms that can enhance the implementation of complexity approaches to study early-stage policy implementation in other contexts.

As shown in Fig. 1, among frameworks, we identified 18 process models, 11 determinant frameworks and 16 evaluation frameworks. Most frameworks (16) are implementation frameworks, including evaluation elements, while 9 frameworks exclusively look at implementation or evaluation. There are 13 descriptive and 18 prescriptive frameworks. A total of 12 frameworks include implementation considerations from the stage of intervention development. Frameworks focus on various evaluation elements, with most frameworks (21) including effectiveness and 16 looking at impact evaluation, 15 at efficacy, 17 frameworks concern process evaluation and 11 related to an evaluation of elements related to the user experience. A total of 3 evaluation frameworks were concerned with evaluation of the planning phase, 8 were concerned with the conducting phase, 11 were concerned with post-execution and 12 were used throughout all stages.

Prominent constructs and steps were extracted from selected frameworks to give an overview of constructs (Additional file 3) and steps (Additional file 4) included in and steps that made part of the frameworks. Constructs that have been mentioned in frameworks include evidence, feasibility, acceptability, etc. and steps reported were moments of the process that the framework suggested carrying out.

### Thematic analysis results

**Fig. 2:**
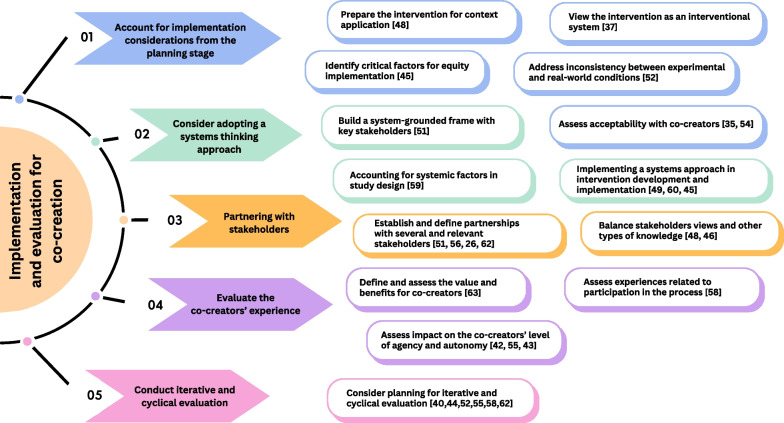
Recommendations

Below, we share findings derived from thematic analysis. Our intention is for these insights to represent recommendations that might be relevant for future research and the implementation of co-creation in practice. Figure 2 represents key themes and sub-themes identified through the thematic analysis of the identified frameworks.

### Early implementation considerations in extracted frameworks

A total of 12 frameworks include reference to early implementation considerations. Eslava-Schmalback et al. [37], recommend identifying critical factors for implementing equity focus recommendations and exploring barriers and facilitators of the intervention from the design phase. Kitson et al. [40] pay attention to preparing the intervention for context application, and Wimbush and Watson [44] call out the possibility of significant inconsistency between an intervention developed in experimental conditions and implementation in the real world.

Among frameworks, Cambon and Alla [33] focus on the context in which the intervention is implemented and argue that this should be viewed as an “interventional system”. In most frameworks, taking into account potential barriers to implementation takes the form of attention to the acceptability of its final users. It is claimed that by assessing its acceptance rate with users, the intervention might address potential barriers to its real-world application. In its description of the intervention cycle, Campbell et al. [31], for instance, advises adopting an iterative process through which the potential recipients’ acceptability of the intervention is assessed and re-examined if needed.

Similarly, Gonot-Schoupinsky and Garip [46] framework dedicates special attention to appropriateness and morality and how the user feels about the intervention as it may impact the intervention’s scalability potential. Assessing acceptability to both end-users and to stakeholders early in the process may be a crucial consideration for large-scale intervention implementation because of its potential to identify potential contextual barrier, enablers and motivations to participation in interventions [46].

### Systems thinking in frameworks

Among the frameworks identified, seven explicitly reference a systems thinking perspective. Best et al. [43] advocate for a systems-grounded frame to be built with key stakeholders. Lo and Karn [41] view complex health programme interventions as systems composed of interdependent features and factors. The latter includes interdependent features and characteristics, such as human behaviours/perceptions, skills and capacity, and governmental and physical structures.

Similarly, Titler [51] recommends finding ways to account for systemic factors in the study design and randomized controlled designs. Zucca et al. [52] place intervention within a systems approach, making a distinction between a complex systems approach, in which variables are so intertwined that the cause and effect relation is uncertain, versus a complicated system, where numerous elements and relationships exist but their relationship can be unveiled and understood. Within the same perspective, Eslava-Schmalback et al. [37] stress the importance of understanding complex systems to advance and enhance implementation.

### Partnerning with stakeholders

Co-creation is considered an approach that promotes engagement in partnership with stakeholders throughout the intervention. We reported on the level of engagement by identifying frameworks that had involved their stakeholders in a partnership, meant, according to Arnstein’s ladder, as the commitment to share planning and decision-making responsibilities through a set structure with its key stakeholders [53].

Six frameworks include elements of partnership with stakeholders. Racher and Annis [48] and Leask et al.’s [25] framework define partnership as the instance in which the stakeholders experience ownership while also (b) providing directional guidance and (c) being invested with responsibilities for activities and outcomes [48].

Partnerships are to be established according to frameworks, with different groups, including with (a) people involved in programme operations, (b) those served or affected by the programme, and (c) primary users of the evaluation [54] or, when community partnerning, between (a) multidisciplinary researchers, (b) the health researchers and community practitioners, and (c) and community health organizations at an international, national and local level [43].

Stakeholders’ views and experience are considered equal to other types of knowledge by Kitson et al. [40] Chen [38], which include patient preferences, views and experience as equally valuable and crucial to evaluate whether an intervention is practical, affordable, suitable, evaluable, and helpful in the real world.

### Evaluating the experience of the co-creators

Benefits of joining the co-creation process might include cognitive, social and personal benefits [55]. To maintain and assert the value of co-creation to the co-creators involved, assessing their experience seems essential.

We reviewed the extent to which frameworks were evaluating the experience of the stakeholder’s involvement in the intervention. Gonot-Schoupinsky and Garip [46] include the assessment of acceptability to include reflections on appropriateness or morality, and how the user might have felt about the intervention, while Jolley et al. [50] suggest investigating barriers to participation and state the importance of ensuring the process is inclusive and values diversity.

Hennesy Lavery et al. [37] and Masso et al. [47], when evaluating the intervention, assess whether the level of agency of participants has increased and regard it as crucial to achieving the sustainability of the intervention. Marckmann et al. [35] stress the importance of evaluating the impact on autonomy while including the elements of health-related empowerment, such as health literacy, respect for individual autonomous choice and protection of privacy and confidentiality.

### Iterative and cyclical evaluation

To comprehensively account for influential implementation elements, iterative evaluation at the planning and conducting phases allows researchers to address and prevent implementation obstacles by assessing the stakeholders’ perceptions and views and adjusting the intervention as needed.

Among frameworks, seven studies recognize the need to perform a more cyclical and iterative evaluation to allow for an intervention to be sustainable within its actual context and replicability to others [36, 44, 47, 50, 54]. Wimbush and Watson, for instance, suggest iterative evaluation as a way to review the intervention's feasibility, practicability, acceptability and for adjusting the programme’s initial design.

## Discussion

This review identified 30 implementation and evaluation frameworks, classified according to their types and according to the categories specified in the data extraction. By analysis the frameworks through thematic analysis, it also offered insights into considerations for when implementing and evaluating future research and practice of co-creation.

Recommendations included accounting for early implementation considerations. Anticipating implementation questions has in fact been argued to be a way to increase the sustainability and maintenance of the intervention in the real-world setting [42] and considered by Moore et al. as crucial for future intervention development and evaluation [28]. Considering the adoption of a systems thinking approach was included as a key facet. Interventions, it is argued, need to be contextualized and understood in, rather than isolated from, the systems they operate within and co-creating interventions with its relevant stakeholders and intended target population, who hold deep knowledge of the systems they are situated within, can ensure a closer tie between theory and context.

By working with multiple levels of influence and with related contextual settings and systems, systems thinking [56]  seems to fit well the scope and intention of co-creation. With its intention to map the larger environment and to identify obstacles and challenges impacting and affecting the public health matter in question, systems thinking enables co-creation to address beyond the isolated causal effect but rather to explore and identify the multiplicity of real-world systematic factors that collate and contribute to the complex problem.

Evaluation is essential and crucial to co-creation and key is formative and cyclical evaluation, as suggested by Anneke van Dijk-de Vries et al. [22]. This, in fact, allows researchers to address and prevent implementation obstacles by assessing the stakeholders’ perceptions and views and adjusting the intervention as needed throughout.

By fostering reflection moments to ensure that end users’ perceptions are continuously captured [25], iterative process evaluation can represent a powerful tool in placing the voice and perception of the co-creators at the core of the intervention cycle. Doing so is particularly relevant when co-creating, as the process and how the co-creators are involved throughout, become part of a co-created intervention’s major outcomes and value in itself  [22].

Partnering with stakeholders and evaluating the co-creators experience is key as the co-created solution is expected to be developed jointly and provide benefits to the co-creators. Valuing the co-creators’ perceived level of co-ownership has been previously regarded essential to the co-creation process [25] and a way to ensure the co-created solution is developed through meaningful engagement.

This review identifies 30 implementation and evaluation frameworks for co-creation and offers recommendations for the planning and evaluating of co-creation for public health. Recommendations emphasize the importance of early implementation considerations, adopting a systems thinking approach, and prioritizing formative and cyclical evaluation. Iterative process evaluation is suggested as a powerful tool to centre co-creators’ voices in intervention cycles, posing and recognizing the value of the co-creation process itself, and not only on the implementation of the co-created solution. To underscore the significance of meaningful engagement and co-ownership when developing co-created solutions, the review highlights attention on the partnering with stakeholders and on an evaluation of the co-creators’ experiences.

This scoping review is conducted as part of the Health CASCADE study and findings will be used to inform the development of further guidance on planning and evaluating co-creation for public health. Authors will dive deeper into the framework by Leask et al. 2019 [25] identified through this review to identify strengths and weakness and expand on the available guidance, through a scoping review, and qualitative interviews with key stakeholders. Authors will also conduct a scoping review on process evaluation studies for co-creation and qualitative interviews with key stakeholders to develop an evaluation framework for co-creation.

## Limitations of the study

Firstly, as we intended to explore the broader implementation field, we included several types of implementation frameworks within our definitions. This meant we captured several non-primary studies, presenting frameworks that had been developed for and/or applied to specific settings and contexts. These studies were later excluded at full-text screening. This might have caused the loss of frameworks that were specific to a context and setting but relevant to the scope of the study. We, however, as previously explained, performed snowballing on the identified frameworks to reduce this possible loss.

Secondly, while we set the search strategy with a specialist librarian, the review might have missed terms used for the same scope by other professionals (e.g. reporting guidelines, checklist or step-by-step how-to).

As part of our analysis, we scoped for implementation intervention development frameworks and did not include public health intervention development frameworks as we were interested in frameworks built to help guide the implementation of the intervention. Therefore this means the search lacks frameworks supporting the intervention development. We applied the search strategy to databases focussing on public health per the review’s scope. Therefore, this review might lack frameworks in databases from social sciences, although the snowballing exercise aimed to reduce the bias as it was performed with no limitations to the databases’ field. It is also worth acknowledging that the frameworks’ classification and data extraction were extracted independently by two reviewers and agreed upon by consensus to ensure the analysis was accurate. Nevertheless, interpretations made as part of the frameworks’ analysis were based on the reviewers’ subjective appraisal.

## Conclusions

This review identified, classified and analysed 30 implementation and evaluation frameworks and offered recommendations for professional stakeholders and researchers wanting to adopt a co-creation approach.

The study recommends, when co-creating, to (a) include implementation considerations from an early stage and at the stage of intervention planning, (b) adopt a systems thinking approach when co-creating, and (c) form a partnership relationship with stakeholders to (d) plan for an iterative and cyclical evaluation and (e) focus on evaluating the co-creators’ experiences.

### Contributions to literature

- This scoping review identifies and classifies 30 implementation and evaluation frameworks for the development, implementation and evaluation of interventions in public health.
- The analysis suggests positioning implementation considerations from an early start of the intervention and adopting a systems thinking approach to the implementation and evaluation of co-created interventions.
- The authors highlight the importance of partnering with stakeholders and recommend carrying out an evaluation that is iterative and cyclical and focusses on the experience of the co-creators.

### Supplementary Information

**Additional file 1. **PRISMA.

**Additional file 2. **Search strategy.

**Additional file 3. **A summary of the constructs found in the frameworks.

**Additional file 4. **A summary of steps found in frameworks.

## Data Availability

Further data and materials are included in the additional files.

## Footnotes

1 https://www.rayyan.ai/
