# Supplementary figures and images for: A review of implementation and evaluation frameworks for public health interventions to inform co-creation: a Health CASCADE study
Source: Health Res Policy Syst. 2024 Mar 28;22:39. doi: 10.1186/s12961-024-01126-6 (PMC10976753; doi:10.1186/s12961-024-01126-6)

**Additional File 1: PRISMA (.pdf)**

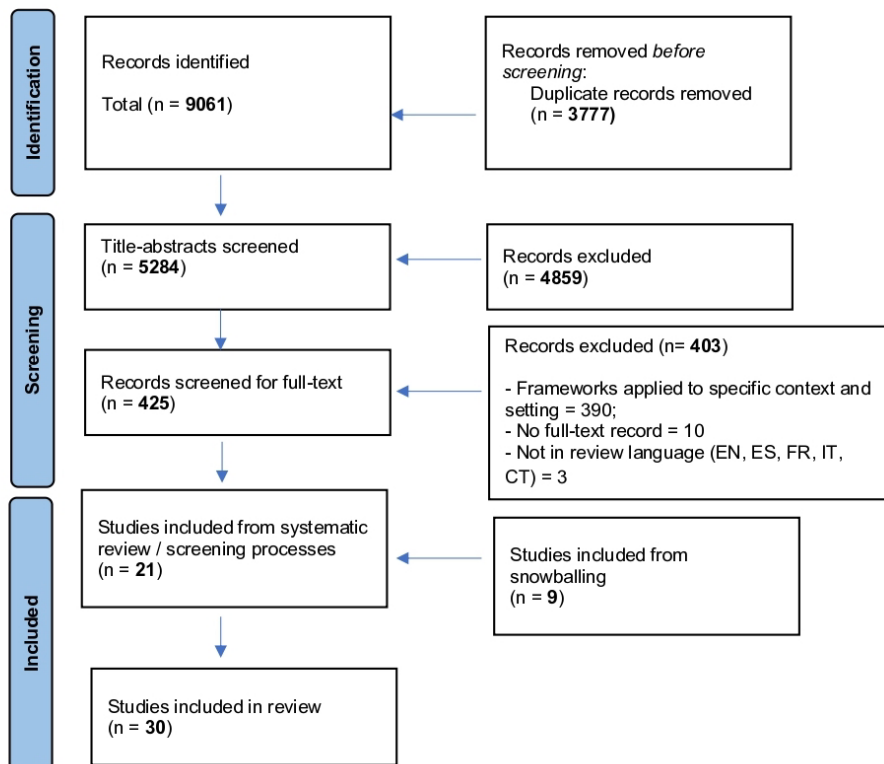

Supplement: Supplementary file 1 — Additional file 1. PRISMA. [file 12961_2024_1126_MOESM1_ESM.pdf]
