# Supplementary material for: A review of implementation and evaluation frameworks for public health interventions to inform co-creation: a Health CASCADE study
Source: Health Res Policy Syst. 2024 Mar 28;22:39. doi: 10.1186/s12961-024-01126-6 (PMC10976753; doi:10.1186/s12961-024-01126-6)
Supplement: Supplementary file 2 — Additional file 2. Search strategy. [file 12961_2024_1126_MOESM2_ESM.pdf]

Additional File 2: Search strategy (.pdf)

Search strategy on PubMed

"Health Plan Implementation"[Mesh]
implement*[tiab]
evaluation[ti]
#1 OR #2 OR #3
"Health Promotion"[Mesh]
policy[ti]
policies[ti]
public health[ti]
health promot*[tiab]
community health[ti]
population health[ti]
health promotion[tiab]
health[ti] AND promot*[ti]
health plan*[ti]
#5 OR #6 OR #7 OR #8 OR #9 OR #10 OR #11 OR #12 OR #13 OR #14
#4 AND #15
framework*[ti]
model[ti]
models[ti]

theor*[ti]

paradigm*[ti]

strateg*[ti]

multidimension*[ti]

perspectiv*[ti]

concept*[ti]

context*[ti]

#17 OR #18 OR #19 OR #20 OR #21 OR #22 OR #23 OR #24 OR #25 OR #26

#16 AND #27

Search strategy on CINAHL

SU health plan implementation OR AB implement*OR TI implement* OR TI evaluation

SU health promotion OR TI policy OR TI Policies OR TI public health OR TI health promot* OR AB health promot* OR TI Community health OR TI population health OR TI health promotion OR AB health promotion OR TI (Health AND promot*) OR TI health plan*

TI framework* OR TI model OR TI models OR TI theor* OR TI paradigm* OR TI strateg* OR TI multidimension* OR TI perspectiv* OR TI concept* OR TI context*
