# Supplementary material for: A review of implementation and evaluation frameworks for public health interventions to inform co-creation: a Health CASCADE study
Source: Health Res Policy Syst. 2024 Mar 28;22:39. doi: 10.1186/s12961-024-01126-6 (PMC10976753; doi:10.1186/s12961-024-01126-6)
Supplement: Supplementary file 3 — Additional file 3. A summary of the constructs found in the frameworks. [file 12961_2024_1126_MOESM3_ESM.pdf]

Additional File 3: A summary of the constructs found in the frameworks (.pdf)

Concept	Definition
Evidence	Evidence is defined by Kitson et al. [53] as codified and non-codified sources of knowledge and includes research evidence alongside practitioner experience, community preferences and experiences, and local information. Cambon et al. [40] propose three ways of categorizing types of data collected to inform the planning and conducting of the intervention (described below). Within we have included relevant extracted constructs from included studies.
Types of evidence	1) Data to explain the mechanisms to be activated [40]. With this scope in mind, Campbell et al. [41] also stress the importance of identifying theory to help build the evidence on whether the intervention might have the desired effect. Lo and Karnon [54] suggest a realist review understand specific aspects as new information is uncovered, and in testing and evaluating the recommendations.
	2) Data to explain the influence of environments, actors and organizations on these mechanisms (determinant of change, existing and effective means of influencing these determinants) [40]. Lo and Karnon [54] propose a qualitative descriptive analysis and integrative reviews to identify and extract data to describe specific barriers/facilitators, what works/does not work.
	3) Data on the feasibility and acceptability of intervention components enabling the intervention inputs to be adjusted [40]. Lo and Karnon [54] include patient and public involvement as a way to ensuring that changes to the health programmes are acceptable, feasible and sustainable. Efficacy is an element reported in Glasgow's et al. framework, intended as the analysis of positive and negative impacts of the conducted intervention alongside behavioral, quality of life, and participant satisfaction outcomes as well as physiologic endpoints. Outcomes to be measured might include the biologic outcome and use but also behavioral outcomes for who deliveres and who received the intervention [46].
Context	Context, according to Kitson et al., is the environment in which the solution is to be implemented and is seen as the interplay of the dominant culture, the leadership roles and the organization's approach to measurement [53].<br><br>Context is the environment or setting in which the proposed change is to be implemented. Context is subdivided into three core elements: an understanding of the prevailing culture, leadership roles and the organization's approach to measurement (evaluation).
Social determinants	Several authors refer to specific social determinants. These are understood as the conditions in which people are born, live and work, and exert sizable effects on the onset and progression of diseases [49]. Best et al. apply the social ecological model as a way to understand of the relationship among diverse environmental and personal factors (individual, organizational and institutional) [39].<br><br>For Eslava-Schmalbach et al. those are the following: Social cohesion, social vulnerability, social exclusion, religion, place of residence, social patterning of individual behaviour, social capital, ethnic/race/culture/language inequalities, sexual behaviour and sexual health, poverty, occupation, life course, social gradient, psychosocial environment at work, food politics, gender/sex [45].
Quality assurance	Covers the principles of participation and partnership as well as the process of programme planning, design and delivery. Is intended as a cyclic process of monitoring and reviewing agreed standards to optimize the likelihood of efficiency and effectiveness [67]. Quality delivery in Carrol et al. concerns whether the intervention is delivered in a way appropriate to achieve what was intended and is seen, for instance, as ongoing monitoring and feedback to those delivering the intervention [42].
Feasibility	Seen as a way to assess the feasibility of the intervention and acceptability to providers and patients [41] and to ensure that overriding practical concerns relating to whether the intervention can work as intended are considered [47]. Chen [43] stresses the importance of assessing real viability concerns, defined as the extent to which the intervention is practical, suitable and acceptable to the real world. Chen [43] highlights the need to evaluate a) whether its implementers can implement it; b) whether it is suitable for implementing organisations to coordinate the related activities, and; c) whether it is affordable, evaluable and e) whether it is clear to the intervention clients and stakeholder that the intervention is solving the problem [43].
Acceptability	An intervention may be feasible but not acceptable in that is not comfortable or satisfying for the user. Acceptability may include notions of appropriatness or morality and be related to how a user feels about the intervention. This consideration, where appropriate, can be anticipated at an early stage of an intervention to facilitate future implementation [47]. Glasgow et al. mention adoption as a way to measure the proportion and representativeness of settings that adopt a given policy or program [46].
Replicability	The extent to which others can replicate the intervention [41], otherwise defined as the potential generalizability of an intervention [40].
Tailorability	The intention to refine and adapt the intervention to practical individual needs so that the design and user instructions are optimised for beneficial impact [40].
Sustainability	Masso et al. [56] mention sustainability as the maintaining of benefits of the program for consumers, in an identifiable form. They stress the importance of the organisational structure and of the building of capacity of the community receiving the program. Glasgow et al. [46] include the notion of maintenance as the extent to which innovations become a relatively stable, enduring part of the behavioral repertoire of an individual (or organization or community), while others refer to the concept as adoption [45].
 Being:<br>Belonging:<br>Becoming:	In Racher and Annis [61] community health actions should be guided by:<br>Interactions as people come together to form a collective unit;<br>Expression by the group of a sense of community;

	Community action by the group: assessing the community, setting goals and planning for change, implementing change, evaluation both the processes carried and the outcomes or changes undertaken [61].
Capacity building	The intention to acquire the skills and resources necessary to assess the health conditions of their community and then plan, implement and evaluate designed to improve these conditions [50]. Also intended at different levels (individuals, organisations and the system). Wimbush et al. [67] intend it as the greater capacity of a community or organization to take action to address health issues in the future.
Reach	According to Glasgow et al [46] refers to the number of people and percentage of the target population affected and the extent to which the individuals reached are representative and include those most at risk.
Dissemination	A purposeful and facilitated process of distributing information and materials to organizations and individuals who can use them to improve health [63].
Facilitation	Defined as a technique by which one person makes things easier for others, describes the type of support required to help people change their attitudes, habits, skills, ways of thinking, and working [53].
**Additional Evaluation elements**	**Description**
Capacity	The assessment of weather the residents have acquired the skills and resources necessary to asses the health conditions of their community and then plan, implement and evaluate accordingly [50].
Potential harm and burdens	Marckmann et al. [55] assess potential harm and burdens and the impact on equity by looking at a) transparency, b) consistency, c) justification, d) participation, e) management conflicts of interest, and f) openness for revision and regulation.
Implementation fidelity	Fidelity was featured as an evaluation element in several frameworks, but was understood differently by the authors. Eslava-Schmalbach et al. [45] view it as the adherence of disadvantaged populations to the equity-focused implementation program or intervention, while Wimbush & Watson [67] present fidelity as composed of the following sub-elements: dose, quality of delivery, participant responsiveness, and program differentiation. Carroll et al. [42] and Gonot-Schoupinsky and Garip [47] describe fidelity as the level of adherence to those who deliver an intervention's intended outline. Carroll et al. [42] include in fidelity the sub-elements of content, coverage, frequency and duration.
Partnership level	Jolley et al. [52] evaluate partnership level by focusing on the following elements: a) at what level did participation occur, b) who had power and control, c) who benefited and how d) what changes were made as a result of the participation. To evaluate the outcome-related success of a partnership, the framework recommends using the criteria of a) an improvement in accessibility of services to users; b) more equitable distribution of services; c) improved efficiency, effectiveness and quality of services along with reduced overlap and duplication; d) improved service experiences for users and carers; and e) improved health status, quality of life and well-being at a population level.
