# Supplementary material for: A review of implementation and evaluation frameworks for public health interventions to inform co-creation: a Health CASCADE study
Source: Health Res Policy Syst. 2024 Mar 28;22:39. doi: 10.1186/s12961-024-01126-6 (PMC10976753; doi:10.1186/s12961-024-01126-6)
Supplement: Supplementary file 4 — Additional file 4. A summary of steps found in frameworks. [file 12961_2024_1126_MOESM4_ESM.pdf]

Additional File 4: A summary of steps found in frameworks (.pdf)

Stage concerned	Steps
Planning	- Identify the population health status [41];<br>- Identify unmet social needs [49];<br>- Validating the theory (data collection and analysis) [40]. To Lewis et al. [77] this is named research phase. In this respect Lo and Karnon [78] include definying research question and searching for inclusion criteria and studies and to conduct a metanalysis;<br>- Defining investigation tools to understand conditions of impact [40];<br>- Situation analysis, problem analysis and needs assessment [60]. According to Green and Kreuter [48] this should include a) a social assessment, b) an epidemiological assessment, c) behavioural and environment assessment, d) an education and ecological assessment, e) an administrative and policy assessment;<br>- Action research, followed by an analysis results of community diagnosis [50];<br>- Elaborating the intervention [40]; Planning the program [37]; Select action or activity and implement [50] and develop goals, objectives and strategies [59];<br>- Considering equity-focused designs [41]; Marckmann et al. [55] suggest specifying and modifying of the normative criteria;<br>- Understanding the contextual and implementation conditions of impact [40].
Conducting	- Wimbush et al. [67] distinguish the intervention implemented whether it's at early start-up, establishement and fully operational;<br>- Skill-based training [50];<br>- Address unmet social needs [49];<br>- Identify and group barriers and facilitators [78]. This according to Damschroder et al. [44] includes understanding the a) characteristics of the intervention, the inner setting, the outer setting, individuals involved, the implementation process;<br>- Conducting the intervention [27]. To Lewis et al. (2016) named research and institutionalization phase. This is done by Implementing equity-focused designs [41]; by action research [50];<br>- Testing an intervention [40];<br>- Process/formative evaluation [60];<br>- Program redesign and re-implementation [60];<br>- Improve outcomes [49];<br>- Maintain and enforce action or activity [50].
Evaluating	- Modelling [41];<br>- Exploratory trial [41];<br>- Definitive RCT [41];<br>- Efficacy evaluation [43];<br>- Impact evaluation [60];<br>- Effectiveness evaluation [43];<br>- Outcome evaluation [60]<br>- Conduct a search for evidence on outcomes and costs [78];<br>- Process evaluation [37,55];<br>- Evaluate population health status [41];<br>- Evaluation of the public health intervention using the specified criteria [55].
Reporting	- Generating recommendations [78] and reporting [27]
